# A Quality Improvement Project to Decrease Fractures Secondary to Metabolic Bone Disease of Prematurity

**DOI:** 10.1097/pq9.0000000000000750

**Published:** 2024-07-29

**Authors:** Nicole M. Rau, Lisa J. Monagle, Ashley M. Fischer

**Affiliations:** From the * Division of Neonatology, Department of Pediatrics, University of Illinois College of Medicine at Peoria, Peoria, Ill.; † Department of Foods and Nutrition, OSF St Francis Medical Center, Peoria, Ill.

## Abstract

**Introduction:**

Osteopenia of prematurity is common in the neonatal intensive care unit, with an incidence of up to 54% in extremely low birthweight infants. The baseline fracture rate in our level IV midwestern neonatal intensive care unit was 13%, with poor compliance with recommended intakes of calcium, calcium:phosphorus ratio, and Vitamin D.

**Methods:**

A multidisciplinary team implemented a screening guideline through four Plan-Do-Study-Act cycles, which addressed staff education, vitamin D screening, and incorporation of calcitriol. In total, 150 patients born between October 1, 2019 and April 30, 2023 were screened for mineral intakes, laboratory abnormalities, and the development of fractures or osteopenia.

**Results:**

The incidence of fractures decreased from 13% to 5.3%. Compliance with mineral intakes improved for calcium, calcium: phosphorus ratio, and Vitamin D. Infants born after the guideline were 4.8 times less likely to develop fractures.

**Conclusion:**

Quality improvement methodology successfully decreased the rate of fractures due to osteopenia of prematurity and increased compliance with recommended mineral intakes.

## INTRODUCTION

### Available Knowledge and Rationale

Metabolic bone disease of prematurity is common in the neonatal intensive care unit (NICU). Other names include osteopenia of prematurity (OOP) or rickets of prematurity, referred to as OOP in the remainder of this article. Estimates of the incidence of OOP vary across studies but are as high as 32% of very low birth weight (<1500 g) and 54% of extremely low birth weight (ELBW, <1000 g) infants.^1,2^ OOP is characterized by radiographic and biochemical changes indicating skeletal demineralization compared with a neonate of similar size and gestation^2^ with frequent laboratory findings of elevated alkaline phosphatase and decreased phosphorus levels. This demineralization may lead to fractures, even with routine handling in the NICU,^3^ and increased incidence of postnatal growth failure in very low birth weight patients.^4^

The cause of OOP is multifactorial but primarily results from the inability to replicate in utero accretion rates of calcium and phosphorus during the third trimester of pregnancy, with most mineral deposition into bone occurring from 24 weeks postmenstrual age (PMA) until term.^5–7^ Over the last month of gestation, approximately 120–150 mg/kg/day of calcium and 70 mg/kg/day of phosphorus is accrued.^8^ This mineral deficit is further exacerbated by inadequate mineral intakes, prolonged total parenteral nutrition (TPN) dependence, immobilization, and medications such as diuretics and steroids.^7^

As survival rates for ELBW patients increase, so does the challenge of preventing related morbidities. Recommendations on optimal enteral mineral intake and serial monitoring of serum phosphorus and alkaline phosphatase were made by the American Academy of Pediatrics (AAP) in 2013.^9^ Despite having nutritional recommendations for the prevention of OOP, prolonged parenteral nutrition, limitations in fluid intake, and intolerance of fortified feeds makes meeting these intakes difficult in the ELBW population. Thus, monitoring for the development of OOP is necessary in the NICU. Various laboratory screening methods have been proposed for at-risk patients beginning at 4–6 weeks of life, typically composed of serum phosphorus and alkaline phosphatase levels at a minimum, with some also recommending serum parathyroid hormone (PTH), 25-(OH) Vitamin D, tubular reabsorption of phosphorus (TRP), urinary calcium: creatinine ratios, and occasionally x-rays or dual x-ray absorptiometry scanning.^2^

### Local Problem and Aims

In our midwestern level IV NICU with 50–60 ELBW admissions per year, our baseline fracture rate during the birth hospitalization was 13% in 2018–2019, with no consistent screening for or treatment of OOP. Our compliance rates with the American Academy of Pediatrics recommended mineral intake at baseline were 80% for phosphorus, 60% for calcium, 60% for optimal calcium/phosphorus ratio, and 27% for Vitamin D. Due to this elevated fracture rate, a quality improvement initiative was established within our division in 2019 to reduce our incidence of OOP by implementing a prevention and screening guideline. A multidisciplinary team was assembled to monitor babies at risk for OOP and provide weekly recommendations. This group also formulated a SMART goal to reduce the incidence of fractures in the ELBW population due to OOP to <10% by December 2021.

## METHODS

### Context

**Fig. 1.**
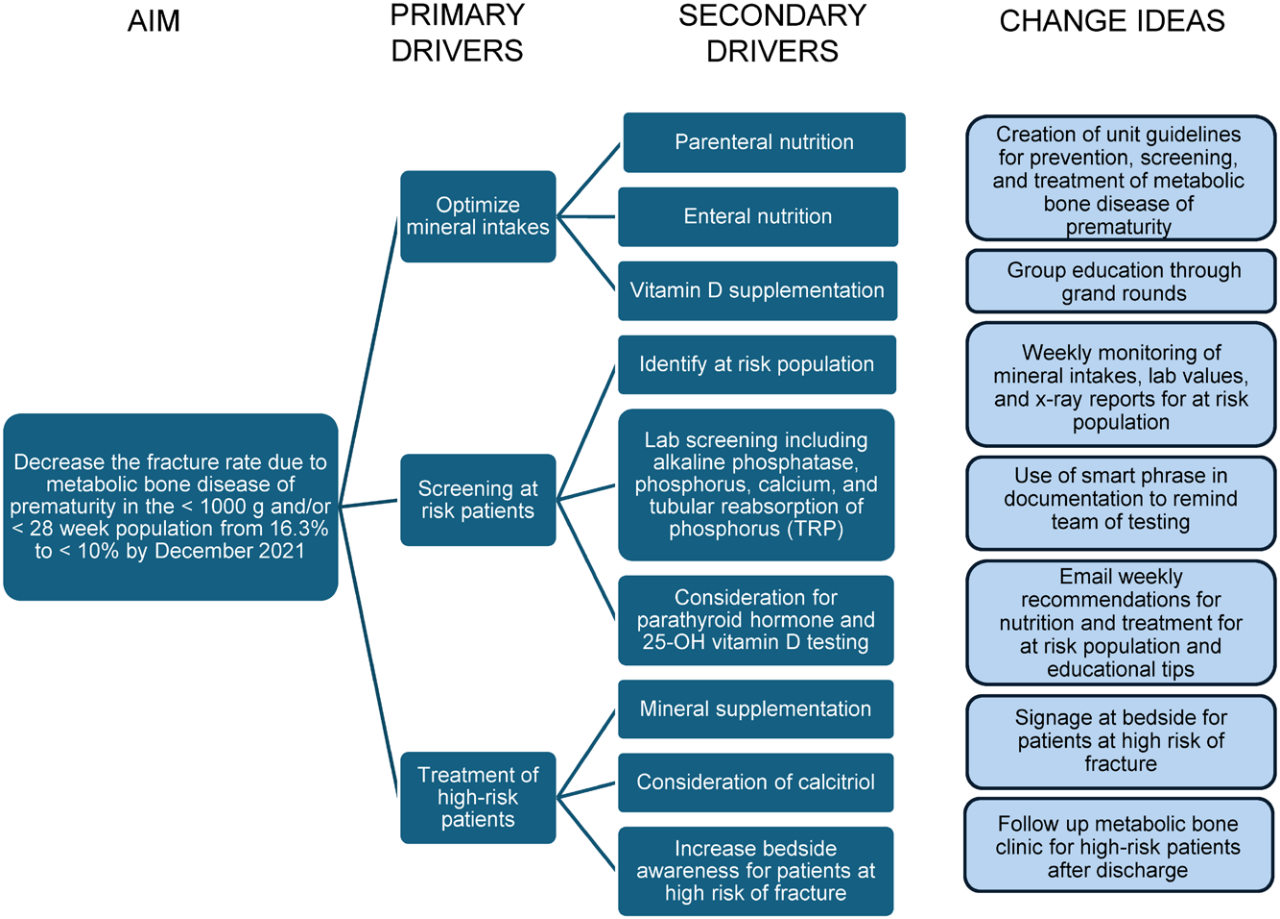
Driver diagram

Three months before the initiation of screening, a multidisciplinary group of neonatologists, nephrologists, endocrinologists, pharmacists (including representatives from the NICU and nutrition support), and dieticians were assembled. A driver diagram (Fig. 1) was formulated with change ideas identified. The group reviewed available literature relating to OOP and developed an evidence-based prevention and screening guideline. This guideline included goals for mineral intakes (both enteral and parenteral; **See table, Supplemental Digital Content 3**, http://links.lww.com/PQ9/A582),^9,10^ laboratory screening criteria, treatment recommendations, and criteria for follow-up after discharge from the NICU. The guideline (**See figure, Supplemental Digital Content 1**, which shows screening algorithm for osteopenia of prematurity, http://links.lww.com/PQ9/A580) was presented to the neonatology group in a monthly meeting with NICU advanced practice nurses and the NICU nursing counsel. Following neonatologist and advanced practice nurses approval, from October 2019 to June 2023, we screened and provided recommendations for 150 infants. One hundred thirty-three were born <28 weeks gestational age, 129 had a birth weight <1000 g, and 64 had TPN use over 4 weeks. The demographics of included infants are presented in Table 1. Laboratory results were routinely obtained, including alkaline phosphatase, phosphorus, urine phosphorus, and urine creatinine levels, to calculate the TRP. PTH and 25-(OH) Vitamin D were obtained if other laboratories were abnormal despite appropriate intakes and supplementation.

**Table 1.** Demographics

Characteristic	Preintervention (55 Patients)	Postintervention (150 Patients)	*P*
Gestational age, wk (mean ± std dev)	28 (±4.2)	26 (±2.7)	0.02
Birth weight, g (mean ± std dev)	1135 (±799)	860 (±383)	0.02
Small for gestational age	14 (25%)	22 (15%)	0.07
Male sex	24 (44%)	70 (47%)	0.70
Days of TPN (mean ± std dev)	41 (±37)	39 (±41)	0.68
Dbili > 2	15 (27%)	34 (23%)	0.49
Use of calcium/phosphorus supplements	17 (31%)	63 (42%)	0.15
Fracture due to osteopenia of prematurity	7 (13%)	8 (5.3%)	0.07*
Use of diuretics >2 wk	29 (53%)	88 (59%)	0.45
Use of steroids (IV or inhaled) >2 wk	31 (56%)	84 (56%)	0.96

* When correcting for GA and BW via logistic regression modeling, risk of fracture due to OOP did reach statistical significance, with a *P* value of 0.04.

### Ethical Considerations

Per the institutional review board, this quality improvement project did not meet the criteria for human subjects’ research, as it was limited to activities designed specifically to improve departmental performance.

## INTERVENTIONS

### Plan-Do-Study-Act (PDSA) Cycle 1: Education and Initiation of Screening Protocol

Education was provided to all providers (physicians, nurse practitioners, dieticians) on goal mineral intakes and ways to optimize intake in enteral and parenteral nutrition, including early addition of phosphorus and calcium in TPN. Additionally, a spreadsheet was created and emailed weekly to all providers to identify infants at risk for OOP based on the guideline inclusion criteria. Initially, group members gathered weekly to complete the spreadsheet via hand calculation of each infant’s parenteral and enteral intakes. Over time, an Excel spreadsheet was created, allowing for more rapid intake calculation. Dieticians kept a living Excel spreadsheet that reported each infant’s intake of calcium, phosphorus, calcium: phosphorus ratio, presence of abnormal laboratories, and diagnosis of fractures or osteopenia identified on routine radiology exams. They emailed this weekly to the two neonatologists. Together, the dieticians and neonatologists provided recommendations on the timing of the next laboratories, utilization of interventions, and optimization of intakes. It was also identified when infants could phase out of testing and which infants needed outpatient follow-up with endocrinology in the metabolic bone clinic. Occasionally, for complex situations or fractures, the team would request an endocrinology consultation be obtained for further assistance. A smart phrase was incorporated into Epic’s problem list (Epic Systems, Verona, Wisc.), our electronic medical record, which used these recommendations. The family advisory council created a fragile bone sign to be used at the bedside of high-risk patients (**See figure, Supplemental Digital Content 2**, which shows bedside fragile bone sign, http://links.lww.com/PQ9/A581). The dietician team also provided verbal feedback to the neonatologists on rounds.

### PDSA Cycle 2: Addition of Vitamin D screening

As part of the newly implemented guideline, PTH and Vitamin D levels were obtained for infants with abnormal alkaline phosphatase and TRP despite appropriate calcium and phosphorus intakes. In January 2020, it was noted that several infants had extremely low 25-(OH) Vitamin D levels. This finding prompted a review of Vitamin D intakes for the screened infants, which showed many infants were not meeting the recommended intake of 400 IU of Vitamin D daily. Vitamin D intakes and recommendations were subsequently included in the weekly screening sheet. Feeding protocols were updated to include adding 200 or 400 IU of Vitamin D when full feeds were achieved to ensure a minimum daily intake of 400 IU.

### PDSA Cycle 3: Updated Screening Guideline

In December 2020, the group reviewed the 80 infants screened since the project began with the OOP guideline and 59 historical patients. It was determined that none of the infants included in either the baseline population or prospectively screened population who were included only due to prolonged diuretic or steroid use had a fracture or were diagnosed with OOP. We, therefore, chose to remove these populations from the screening guideline and focus solely on infants born <28 weeks PMA, with a birthweight <1000 g, or with TPN >4 weeks. These infants were also excluded from the overall results.

### PDSA Cycle 4: Incorporation of Calcitriol

In January 2021, the group attended a webinar held by the Children’s Hospital of Philadelphia on metabolic bone disease of prematurity, which discussed the use of calcitriol in cases of persistently elevated PTH. The article published by this group was also reviewed,^11^ and the guideline was updated to include consideration for calcitriol in cases of hypophosphatemia and elevated PTH if no improvement was noted after 2 weeks of adequate calcium and phosphorus intake.

## STUDY MEASURES

Eligible neonates born from October 1, 2019, through February 28, 2023, were identified in real-time by NICU dieticians and were included in the analysis if the patient survived >4 weeks. Baseline data were collected retrospectively on infants born between January 1, 2018, and September 30, 2019, and survived >4 weeks. Retrospective data included fractures, days of high-risk medications (including diuretics, TPN, steroids), growth data, laboratory and x-ray data, and measurement of mineral intakes weekly from DOL 7–28 and every 2 weeks. These data determined historical compliance with recommended mineral intakes and historical fracture rates.

Neonates born after October 1, 2019, were considered part of the postimplementation group. The process measures for this project included the percentage of infants receiving goal intakes of calcium, phosphorus, Vitamin D, goal calcium: phosphorus ratio, the rate of laboratory values outside of target ranges, and types of recommendations made by the QI team. Measurements through April 30, 2023 are included in this article. Outcome measures included new fractures due to OOP and a new diagnosis of OOP from January 1, 2018, through April 30, 2023. Osteopenia and fractures were identified by reviewing the radiology report for every x-ray obtained during the admission, including those obtained for clinical symptoms of a fracture and those obtained for other indications like line or tube placement. The balancing measure chosen was the percentage of infants on calcium or phosphorus supplementation, as additional supplements would complicate the medication routine, increase the volume of medicines given at one time, possibly resulting in emesis, and are known to cause GI upset. Originally, nephrocalcinosis was monitored as a balancing measure; however, this was not observed during the initial year of monitoring, so it was subsequently dropped.

## ANALYSIS

Process and balancing measures were plotted on p charts. Outcome measures were plotted on t-charts due to the infrequent nature of these events. Excel with QI Macros was used to generate all charts. Standard rules were used to analyze special cause variation. Statistical analysis comparing the historical and postintervention populations was done using chi-square, t test, analysis of variance testing with post hoc analysis, and binary logistic regression using IBM SPSS statistics software, version 29.

## RESULTS

### Demographics

Baseline data included 59 patients surviving > 4 weeks in the NICU: 43 met gestational and/or birth weight criteria, 12 met the criteria of TPN > 4 weeks, and four met the criteria for steroids or diuretics for >2 weeks. As the steroid and diuretic criteria were dropped in 2020, these four patients were not included in the analysis, and none developed a fracture. Postintervention patients included those born after October 1, 2019, with 150 patients meeting the criteria for gestational age, birth weight, and/or TPN duration >4 weeks. Patients in the postintervention phase were significantly younger, on average, by 2 weeks and smaller, by 275 g (Table 1).

### Primary Outcomes

**Fig. 2.**
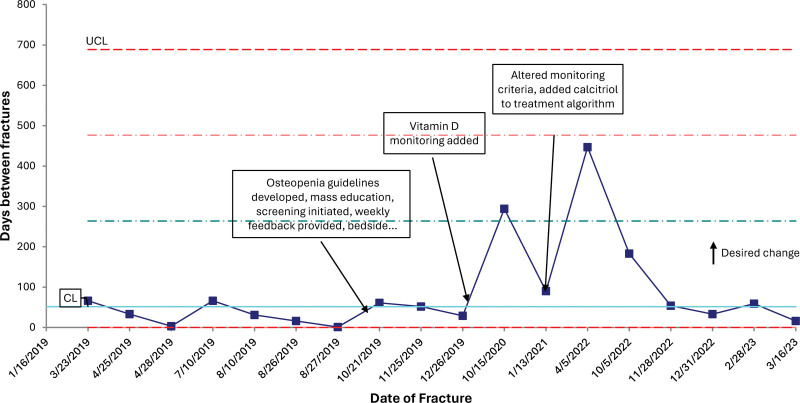
T-chart showing days between fractures due to OOP. The baseline is 51 days between fractures.

The fracture rate in the historical population was 13% and 5.3% in the postintervention phase. As depicted on the t-chart (Fig. 2), between December 2019 and November 2022, there were more days than predicted between fractures based on the historical population. Starting in November 2022, multiple extremely premature infants requiring prolonged periods of TPN were born, and one patient suffered from two fractures, bringing the period between fractures back down to the baseline of 51 days. No fracture occurred before the initiation of screening at 4 weeks of age.

Given the historical population differed significantly from the postintervention population, logistic regression was performed to ascertain the effects of gestational age, birth weight, and the quality improvement project on the likelihood of fracture due to OOP. The logistic regression model was statistically significant, X^2^(5) = 37.1, *P* < 0.001. The model explained 40.6% (Nagelkerke R^2^) of the variance in fractures due to OOP and correctly classified 92.7% of cases. Infants born after the quality improvement project were 4.8 times less likely to have a fracture due to OOP. Decreasing gestational age and birth weight were significantly associated with an increased fracture risk in this model.

### Process Measures

**Fig. 3.**
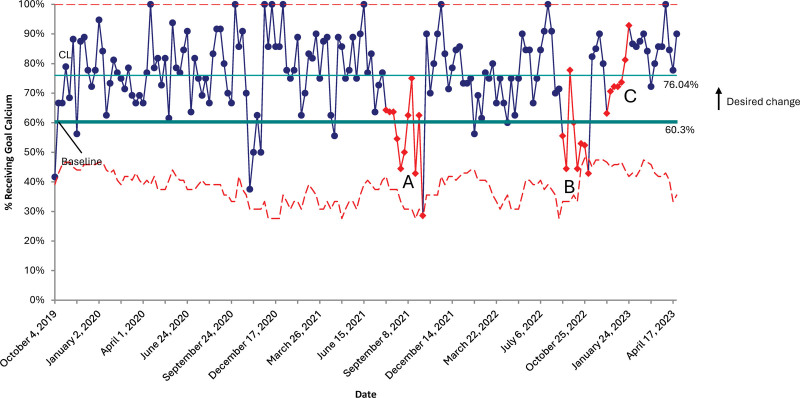
P-chart representing the percentage of patients receiving goal calcium intakes. A: Special cause variation is due to three ELBW infants with hypophosphatemia in the first month of life with reduced calcium/phosphorous ratio to maximize phosphorous content. B: Special cause variation due to two infants with hypercalcemia, one infant with hypophosphatemia, and two infants with fluid restriction limiting calcium intake to just below goal. C: Special cause variation due to multiple infants with fluid restriction and on TPN.

**Fig. 4.**
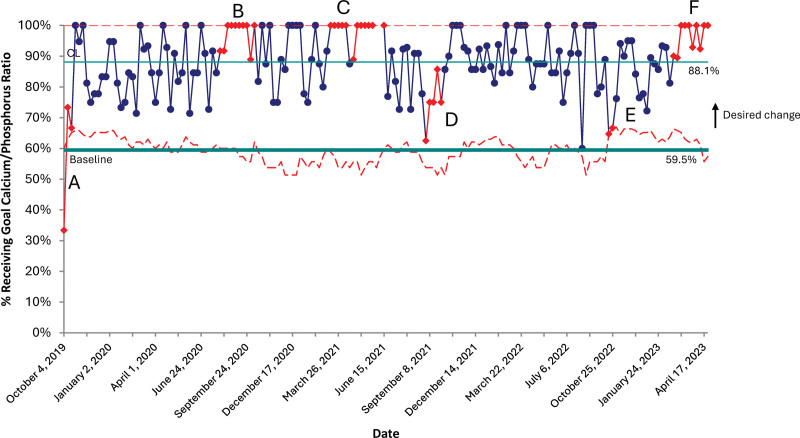
P-chart representing the percentage of patients receiving goal calcium. Phosphorus ratio. A: Special cause variation due to nonadherence to guidelines in the first month of implementation. B/C: Special cause variation due to decreased TPN usage with increased enteral feeds, which provide correct calcium-to-phosphorus ratios. D/E: special cause variation due to multiple infants with hypophosphatemia and reversed calcium-to-phosphorus ratios to maximize phosphorus mineral content in TPN. F: Special cause variation is due to decreased TPN usage with increased enteral feeds, which provide correct calcium-to-phosphorus ratios.

**Fig. 5.**
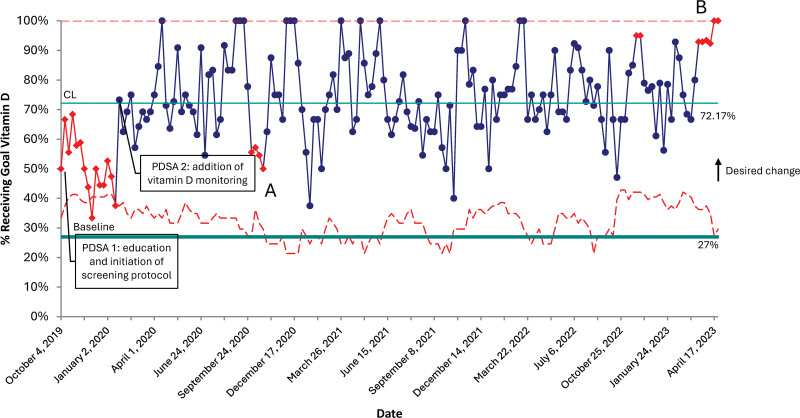
P-chart representing patients receiving goal Vitamin D intakes. A, Special cause variation is due to multiple ELBW infants on TPN, which limits the Vit D content. B, Special cause variation is due to the high number of infants on full enteral feeds with appropriate vitamin D supplementation.

There was a significant improvement in all process measures (except for phosphorus intake, which was 80.7% at baseline) sustained through the initiative. Within the first month of the project, intakes for calcium and optimal calcium: phosphorus ratios significantly improved over baseline values, from 60.4% to 76.0% for calcium and from 59.5% to 88.1% for the calcium: phosphorus ratio. Vitamin D intake improved modestly from the historical population during the first PDSA cycle. Still, it improved dramatically after the second PDSA cycle from 27.0% at baseline to 72.2% in PDSA cycle two and was sustained (Figs. 3–5).

Laboratory values were considered outside of range if alkaline phosphatase was >500 U/L, serum phosphorus was <5.5 or >9.0 mg/dL, TRP was >95%, or 25-OHD was <20. The most frequent laboratory outside range was a low phosphorus level, which occurred ~28% of the time. Elevated alkaline phosphatase was found ~25% of the time and an elevated TRP 10% of the time.

Recommendations from the QI team included multiple categories: TPN changes (44%), addition or adjustment of calcium or phosphorus supplements (21.4%), signage at the bedside (18.9%), increased Vitamin D dosage (11.2%), enteral nutrition changes (1.9%), initiation of calcitriol (1.5%), and endocrinology consultation (1.1%). Initially, the most frequent recommendations were TPN optimization and initiating supplements. However, as the initiative progressed, using bedside signage and adding calcitriol became more frequent recommendations.

### Balancing Measures

Calcium or phosphorus supplementation varied significantly over time, with a baseline between 5% and 24% of the screening population. The level of supplementation did not increase consistently over time. It did not correlate with fractures, and as the project progressed, supplements were often initiated before team recommendations. No nephrocalcinosis developed in infants receiving calcium or phosphorus supplementation.

## DISCUSSION

QI methodologies effectively reduced the incidence of fractures due to OOP from a baseline of 13% to 5.3% over 3.5 years. Although guidelines and recommendations for optimizing bone health are present in the literature, very few other QI projects discussing the prevention of fractures due to OOP have been published.^12,13^ This article adds significantly to the clinical translation of those guidelines to the bedside. Additionally, many screening protocols rely on alkaline phosphatase alone. However, our results suggest that adding phosphorus and TRP to the screening protocol may improve the identification of affected infants.

Effective strategies included staff education, weekly monitoring, and optimization of mineral and vitamin D intakes, and scheduled laboratory monitoring for at-risk populations. We could not eliminate the occurrence of fractures, as some did occur for the ELBW infants on prolonged periods of TPN. Over time, the process of monitoring and providing team feedback has been streamlined. It typically takes a dietician about 2 hours and a neonatologist about 15 minutes per week, allowing the initiative to be sustained.

The team used similar interventions described in the articles by Krithika et al and Cromwell et al^12,13^ It was standard in the unit before the initiative to fortify feeds with human milk fortifier at 100 mL/kg/day and to initiate calcium immediately in TPN; however, during the initiative, we added phosphorus to TPN on day of life one as well. Our QI initiative also added monitoring of mineral intakes and Vitamin D and assisting the clinical team in interpreting laboratory results and trends. Although the recommendations for calcium and phosphorus intake were not new, there was a general lack of awareness of mineral and Vitamin D intakes, as evidenced by the suboptimal compliance with Vitamin D recommendations. Finally, patients requiring elemental formulas and those needing severe fluid restriction due to chronic lung disease were largely unable to meet recommended intakes without the addition of supplements. These recommendations were unobtainable for patients on only TPN at >2 weeks of age.

The incidence of fractures did increase in the last year of the initiative with the birth of multiple 22- to 24-week infants who required intestinal surgery and were on TPN for prolonged periods. These patients also likely contributed to the demographic differences between baseline and postintervention populations. TPN cholestasis was found in five of the eight patients experiencing a fracture after project onset. Metabolic bone disease is a well-known complication for both children and adults with cholestasis.^14,15^ As Intralipid 20% (Fresenius Kabi, Bad Homburg, Germany) is currently the standard parenteral fat in our institution and cholestasis is commonly encountered with its use, this is a potential opportunity. SMOFlipid (Fresenius Kabi, Bad Homburg, Germany) is an alternative parenteral fat that has been increasingly used in the neonatal population within the United States and may be beneficial for the prevention of cholestasis and OOP.^16,17^

This project has several limitations. First, multiple interventions were included at the beginning of the QI project; thus, it is difficult to determine the impact of individual components on outcomes. Secondly, our institution uses a 3-in-1 system to deliver parenteral nutrition, which may result in suboptimal mineral delivery to our neonates due to stability concerns at higher calcium and phosphorus concentrations.^18^ This led to suboptimal intakes for our infants on parenteral nutrition, which may not be a problem in units that use other parenteral nutrition delivery systems. Finally, we did not perform routine radiographic examinations to assess for fracture unless a patient experienced clinical symptoms. Thus, there is a possibility some fractures may have occurred and remained undetected.

## CONCLUDING SUMMARY

Our quality improvement project, which incorporated standardized guidelines, weekly team feedback, and utilization of bi-weekly screening laboratories to guide mineral supplementation, decreased the number of fractures due to OOP in our 60-bed level IV NICU. Other facilities can replicate this work even if they do not have a pediatric endocrinologist readily available. Further steps include reducing the incidence of cholestasis and considering the impact of different parenteral lipid solutions.

## ACKNOWLEDGMENTS

The authors would like to acknowledge the contributions of Angela Lollock, RD, and Douglas Drenckpohl, RD, who contributed to guideline development and the collection of weekly data.
